# An Antibacterial Strategy of Mg-Cu Bone Grafting in Infection-Mediated Periodontics

**DOI:** 10.1155/2020/7289208

**Published:** 2020-08-28

**Authors:** Xue Zhao, Peng Wan, Hongyan Wang, Shuwei Zhang, Jingbo Liu, Chunrong Chang, Ke Yang, Yaping Pan

**Affiliations:** ^1^Department of Periodontics, School and Hospital of Stomatology, China Medical University, Liaoning Provincial Key Laboratory of Oral Disease, Shenyang, Liaoning 110002, China; ^2^School of Materials Science and Engineering, Dongguan University of Technology, Dongguan, Guangdong 523808, China; ^3^Institute of Metal Research, Chinese Academic of Sciences, Shenyang, Liaoning 110016, China; ^4^Department of Oral Biology, School and Hospital of Stomatology, China Medical University, Liaoning Provincial Key Laboratory of Oral Disease, Shenyang, Liaoning 110002, China

## Abstract

Periodontal diseases are mainly the results of infections and inflammation of the gum and bone that surround and support the teeth. In this study, the alveolar bone destruction in periodontitis is hypothesized to be treated with novel Mg-Cu alloy grafts due to their antimicrobial and osteopromotive properties. In order to study this new strategy using Mg-Cu alloy grafts as a periodontal bone substitute, the *in vitro* degradation and antibacterial performance were examined. The pH variation and Mg^2+^ and Cu^2+^ release of Mg-Cu alloy extracts were measured. *Porphyromonas gingivalis* (*P. gingivalis*) and *Aggregatibacter actinomycetemcomitans* (*A. actinomycetemcomitans*), two common bacteria associated with periodontal disease, were cultured in Mg-Cu alloy extracts, and bacterial survival rate was evaluated. The changes of bacterial biofilm and its structure were revealed by scanning electron microscopy (SEM) and transmission electronic microscopy (TEM), respectively. The results showed that the Mg-Cu alloy could significantly decrease the survival rates of both *P. gingivalis* and *A. actinomycetemcomitans*. Furthermore, the bacterial biofilms were completely destroyed in Mg-Cu alloy extracts, and the bacterial cell membranes were damaged, finally leading to bacterial apoptosis. These results indicate that the Mg-Cu alloy can effectively eliminate periodontal pathogens, and the use of Mg-Cu in periodontal bone grafts has a great potential to prevent infections after periodontal surgery.

## 1. Introduction

Periodontal disease (PD) is one of the most common diseases in humans. Approximately 750,847 million people are suffering from PD worldwide according to the global burden of disease study in 2016 [1, 2]. It is characterized by progressive destruction of the tooth-supporting apparatus [3], especially loss of alveolar bone, which is naturally irreversible. Therefore, periodontal bone grafting, a medical device promoting periodontal bone regeneration, has been widely used in periodontal regeneration surgery [4].

Nowadays, numerous bone grafts are used in periodontal surgeries, showing good biocompatibility and osteogenesis. Autogenous bone graft, as the gold standard bone graft currently, often requires a second invasive surgical procedure on the donor site. However, the limited source and volume of the harvested bones and unpredictable resorption rate among patients are main shortcomings. Alternatives to autografts are allografts and xenografts, but they may cause disease transmission and bring risks of infection [5]. The limitations of the above two approaches can be overcome by the use of synthesized bone grafts. Recently, biodegradable magnesium (Mg) alloy implants have received tremendous attention, since they have demonstrated good biocompatibility and similar mechanical properties to natural bone [6, 7]. Compared with other traditional metallic bone implants, the elastic modulus of Mg is much closer to the natural bone [8]. What is more, Mg has an ideal desorption rate and can be gradually consumed or excreted in the human body and then disappear after the healing of bone tissues [9–11].

Mg alloys have been reported to have an antibacterial ability due to the large amount of OH^−^ produced during degradation resulting in a strong alkaline environment [12–15]. However, high pH can be balanced in body fluid when a Mg alloy device is implanted, and hence, the antibacterial ability will be diminished. Thus, it can be meaningful to explore the possibility of adding a proper metal element with antibacterial effect in magnesium.

Copper (Cu) has been found to have antibacterial ability since 1885 [16]. Nowadays, Cu has been added into medical metal materials to offer antimicrobial activities in stainless steels [17, 18], titanium alloys [19], and cobalt-based alloys [20]. Our previous studies have demonstrated the biocompatibility and osteogenic promotion of the Mg-Cu alloy as a biodegradable bone implant [21, 22]. Many studies have proven that addition of Cu in implant material could both kill bacteria and stimulate biological responses to mesenchymal stem cells like proliferation and osteogenic differentiation [23]. These properties make the Mg-Cu alloy an attractive implant material for preventing infections.

Meanwhile, the oral environment is known for the coexistence of various bacteria, with more than 300 distinct bacterial species in the human gingival crevice. It has been proven that periodontal bacteria exist as biofilms composed of very complex mixtures of bacterial species, and their formation is highly ordered. Streptococci such as *Streptococcus gordonii* (*S. gordonii*) represent the primary colonizing bacteria which can adhere to saliva-coated teeth and gingiva surfaces and provide a base for subsequent colonization of other bacterial species [24]. *Fusobacterium nucleatum* (*F. nucleatum*) plays a role as a bridging organism connecting early and late colonizing bacteria such as *Porphyromonas gingivalis* (*P. gingivalis*) and *Aggregatibacter actinomycetemcomitans* (*A. actinomycetemcomitans*) [25]. *P. gingivalis* is known as a keystone bacteria in periodontal diseases as a part of the red complex of periodontal pathogens [26–28]. *A. actinomycetemcomitans* is regarded as the main periodontopathogen in aggressive periodontitis and peri-implantitis [29–31]. Both *P. gingivalis* and *A. actinomycetemcomitans* are capable of invading the gingival epithelial cell and then produce virulence factors to disrupt host-microbial homeostasis, resulting in inflammation and bone loss.

At present, no clinically used natural or synthesized grafts display antibacterial ability. Periodontists thus rely heavily on the myriad antibiotic administration to prevent postoperative infections, leading to a worldwide antibiotic abuse problem [32]. For this reason, an antibacterial bone graft which is unlikely to cause the development of drug resistance is undeniably beneficial [33, 34].

Based on the primary studies, we supposed the biodegradable Mg-Cu alloy as a grafting material to fill the infrabony pocket of periodontal bone resorption. The objective of this study was to explore the antibacterial abilities of Mg-Cu against periodontal anaerobic bacteria in order to provide more evidence for clinical application of this novel material.

## 2. Material and Experimental Methods

### 2.1. Material Preparation

Three Mg-Cu alloys with gradient contents of 0.1, 0.2, and 0.4 wt.% Cu were prepared by conventional melting technique, which were assigned as Mg-0.1Cu, Mg-0.2Cu, and Mg-0.4Cu, respectively. The Mg-Cu alloys were cut into disks with size of *φ*10 mm × 3 mm, grounded and polished with SiC papers, and finally ultrasonically cleaned with acetone and ethanol. Pure Mg and *β*-TCP (*β*-tricalcium phosphate) were used as control groups.

### 2.2. Material Extract Preparation

Extracts of the Mg-Cu alloys were prepared according to ISO 10993-5. The ratio of surface area to extraction medium was 1.25 cm^2^/mL. Samples were immersed in brain heart infusion (BHI) broth in a humidified atmosphere with 5% CO_2_ at 37 ± 0.5°C for 3 h, 6 h, 12 h, 24 h, 48 h, and 72 h. Then, the extracts were filtered and refrigerated at 4°C.

### 2.3. pH and Ion Release Measurements

The pH value of extracts was measured at intervals. Concentration of Mg^2+^ and Cu^2+^ in the extracts was measured using ICP-OES at each time point. The measurements were performed in triplicate.

### 2.4. In Vitro Antibacterial Effect Evaluation

#### 2.4.1. Bacterial Strain Preparation

The bacterial strains used for the present study were *Streptococcus gordonii* Challis CH1 (*S. gordonii*), *Fusobacterium nucleatum* ATCC25586 (*F. nucleatum*), *Porphyromonas gingivalis* ATCC 33277 (*P. gingivalis*), and *Aggregatibacter actinomycetemcomitans* ATCC 43718 (*A. actinomycetemcomitans*), which represent the early, middle, and late colonizers of the bacterial accretion in the periodontal plaque biofilm. All the bacteria in the test were obtained from the Department of Oral Biology, School of Stomatology, China Medical University.

Bacterial strains of *P. gingivalis*, *A. actinomycetemcomitans*, and *F. nucleatum* were cultured in a freshly prepared brain heart infusion (BHI) agar plate (Difco Laboratories, MI) supplemented with 5% sterile defibrinated sheep blood, 1% hemin, and 0.1% menadione in a chamber under anaerobic conditions of 80% N_2_, 10% H_2_, and 10% CO_2_. *S. gordonii* was cultured aerobically in freshly prepared BHI agar plate supplemented with 1% yeast extract for 16 h at 37°C.

All the bacterial suspensions for the test were suspended in a BHI broth until reaching a final density of 1 × 10^7^ colony forming units (CFU)/mL.

#### 2.4.2. Bacterial Concentration and Living Rate Measurements

The antibacterial activity was determined using the indirect method by culturing bacteria in the Mg-Cu alloy extracts. Tested bacteria were diluted by extracts into 1 × 10^5^ CFU/mL, which were then cultured in anaerobic conditions for 24 h. The BHI broth was served as the blank group. The concentration of extracts cultured with bacterial suspensions was determined by real-time PCR assay. The number of living bacteria was determined by the bacterial counting method. The bacterial suspensions were diluted to 1 × 10^3^ CFU/mL, 0.1 mL of which was spread on nutrition agar plates evenly, followed by further incubation at 37°C for 72 h before counting the bacteria colonies. All the tests were conducted three times.

#### 2.4.3. Live/Dead Staining

Tested bacteria were diluted by extracts into 1 × 10^5^ CFU/mL, which were then cultured in anaerobic conditions for 24 h. The bacterial biofilms attached on the base of the plates were fixed by 1% paraformaldehyde for 1 h and then stained with 2.5 *μ*L/mL propidium iodide (PI) for 30 min. The viability of the bacteria was examined with florescence microscope.

#### 2.4.4. SEM Analysis

Tested bacteria were diluted by extracts into 1 × 10^5^ CFU/mL, which were then cultured in anaerobic conditions for 24 h. The bacterial biofilms attached on the base of the plates were fixed with 2.5% glutaraldehyde in PBS (pH = 7.4) for 1 h at room temperature, then washed with PBS for three times and gradually dehydrated with ethanol. The processed samples were smeared onto a copper plate followed by gold sputtering, and images were acquired on a scanning electron microscope (SEM, Inspect F50, FEI Company, USA).

#### 2.4.5. TEM Analysis

Tested bacteria were diluted by extracts into 1 × 10^7^ CFU/mL, which were then cultured in anaerobic conditions for 24 h. The cocultured bacterial suspensions were centrifuged at 5000 rpm for 20 min, and the bacteria were fixed with 2.5% glutaraldehyde. After dehydration with ethanol, the bacteria were embedded with LR white medium (EMS, PA), which was then cut into sections. The sections were contrasted with uranyl acetate and were examined under a transmission electron microscopy (TEM, H7650 Hitachi, Japan).

## 3. Results

### 3.1. pH Variation and Ion Release

**Figure 1:**
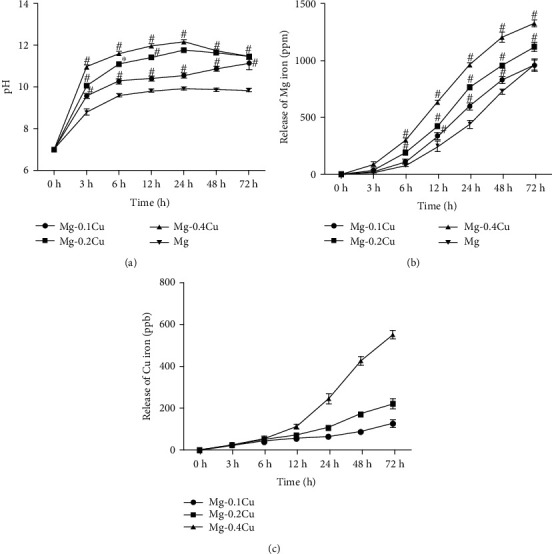
(a) pH value of the Mg-Cu alloy and Mg extracts. The results are in line with the immersion time. (b) Mg^2+^ concentration in the Mg-Cu alloy and Mg extracts and (c) Cu^2+^ concentration in Mg-Cu alloy extracts. The concentration of Cu^2+^ was positively related to the Cu content in the Mg-Cu alloy. The Mg-0.4Cu alloy released much more Cu^2+^ than the other Mg-Cu alloys. # refers to a significant difference between Mg-Cu alloy groups and the Mg group (*P* < 0.05, *n* = 3).

Figure 1(a) shows the pH values of the medium immersed with Mg-Cu alloys compared with pure Mg. The results were in line with the immersion time. The pH value was increased with the increase of Cu content in the Mg-Cu alloy, indicating higher degradation rate. For all the Mg-Cu groups, the pH values increased quickly in the first 6 h and then remained stable above 10 afterwards.

The release of Mg^2+^ and Cu^2+^ after immersion is plotted in Figures 1(b) and 1(c). The higher the pH value was, the more the Mg^2+^ and Cu^2+^ released from Mg-Cu alloy extracts. For the Mg-0.1Cu alloy, the concentration of Mg^2+^ showed no significant difference with pure Mg at 72 h. As the concentration of Cu^2+^ was positively related to the Cu content in the alloy, the Mg-0.4Cu alloy released much more Cu^2+^ than the other Mg-Cu alloys.

### 3.2. Bacterial Survival

**Figure 2:**
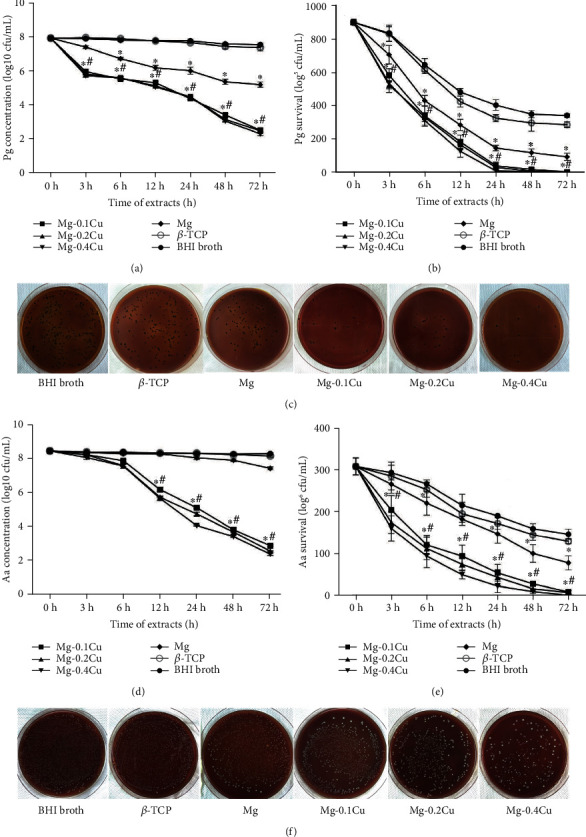
The responses of *P. gingivalis* (*Pg*) to the Mg-Cu alloy and Mg and *β*-TCP extracts: (a) bacterial concentration; (b) live bacterial colonies; (c) optical images of the bacterial colonies in nutrition agar plates and the responses of *A. actinomycetemcomitans* (*Aa*) to the Mg-Cu alloy and Mg and *β*-TCP extracts; (d) bacterial concentration; (e) live bacterial colonies; (f) optical images of the bacterial colonies in nutrition agar plates. ∗ refers to a significant difference between Mg-Cu alloy groups (Mg-0.1Cu, Mg-0.2Cu, and Mg-0.3Cu) and the BHI broth and *β*-TCP groups. # refers to a significant difference between Mg-Cu alloy groups and the Mg group (*P* < 0.05, *n* = 3).

One of the most attractive behaviors of the Mg-Cu alloy system is its remarkable antibacterial activity. In this study, *P. gingivalis* and *A. actinomycetemcomitans*, two common bacteria associated with PD, were used to evaluate the antibacterial activity of the Mg-Cu alloy for bone graft application. As shown in Figure 2, Mg-Cu alloys demonstrated significant inhibition of growths of both *P. gingivalis* (Figures 2(a)–2(c)) and *A. actinomycetemcomitans* (Figures 2(d)–2(f)) (*P* < 0.05). The *β*-TCP group did not show any antibacterial effect as expected. A series of extracts were prepared by incubating samples with bacterial culture medium for different time intervals. For pure Mg and Mg-Cu alloy groups, both the bacterial concentration (Figures 2(a) and 2(d)) and the live bacterial colony (Figures 2(b) and 2(e)) were decreased with increase of incubation time. It should also be noted that the bacterial concentration and colony number for Mg-Cu groups were always less than those of pure Mg, indicating that the release of Cu^2+^ further enhanced the antibacterial ability of Mg. But the three Mg-Cu alloy groups did not show difference in both bacterial concentration and live bacterial colony. *P. gingivalis* and *A. actinomycetemcomitans* reacted differently to the extract of Mg-Cu alloys. For *P. gingivalis*, the inhibition started to become noticeable in the extract with 3 h incubation; however, for *A. actinomycetemcomitans*, the same effect started in the extract after 12 h incubation. The optical images of the *P. gingivalis* (Figure 2(c)) and *A. actinomycetemcomitans* (Figure 2(f)) in nutrition agar plates show the similar results, i.e., for pure Mg and Mg-Cu groups, the numbers of bacterial colonies were much smaller than those of the control group.

### 3.3. Confocal Fluorescence Microscope Analysis

**Figure 3:**
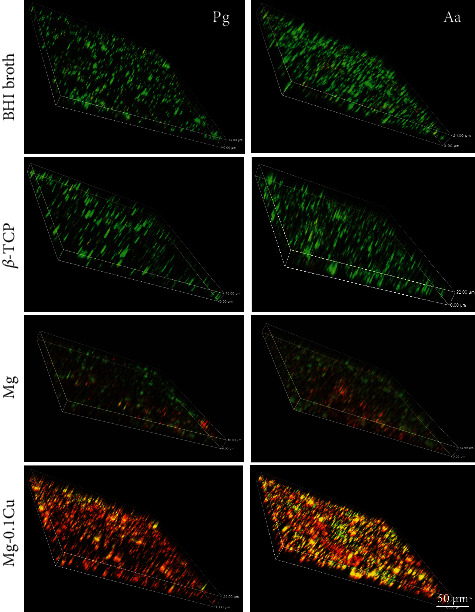
Live/dead imaging of Pg and Aa after culturing in Mg-0.1Cu extracts for 24 h under a confocal fluorescence microscope. The green color indicates live bacterial while the red color indicates dead bacterial. Majorities of both Pg and Aa were dead in the Mg-0.1Cu alloy extract after culturing for 24 h.

The activities of *P. gingivalis* and *A. actinomycetemcomitans* single-species bacterial biofilms were assessed by live/dead staining (Figure 3). A confocal fluorescence microscope was used to image both *P. gingivalis* and *A. actinomycetemcomitans* after incubation with the Mg-Cu alloy extract for 24 h. For the blank and *β*-TCP (positive) groups, amounts of live bacteria were observed, showing a thick biofilm compared with the pure Mg group. Approximate fifty-fifty live and dead bacteria were found in the pure Mg group. However, for the Mg-0.1Cu group, only few live bacteria could be found.

### 3.4. Scanning Electron Microscope Analysis

**Figure 4:**
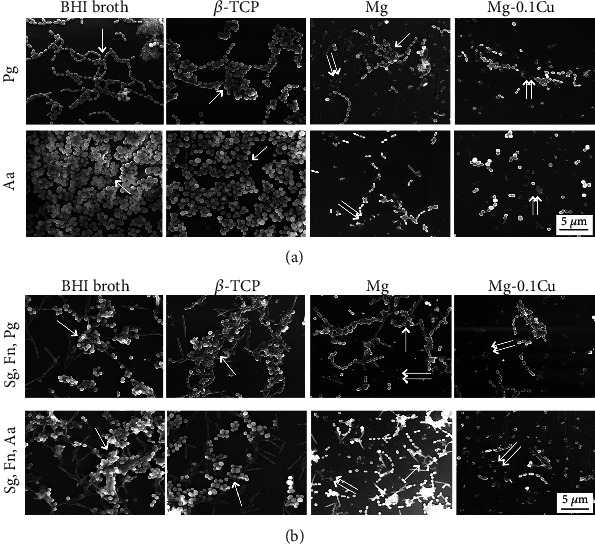
SEM images of bacterial biofilm after culturing in the extract for 24 h. (a) Single-species biofilm of Pg and Aa, (b) multispecies biofilm of Sg, Fn, and Pg and Sg, Fn, and Aa, scale bar = 5 *μ*m. Single arrow shows the normal bacterial biofilm. Double arrows show the damaged bacterial biofilm. The bacteria in the Mg-0.1Cu alloy group lost connection with each other, and the biofilm collapsed completely due to death of bacteria.

To further explore the antibacterial mechanism of Mg-Cu alloys, the status of the bacterial biofilm was assessed by SEM (Figure 4). For single-species biofilms of *P. gingivalis* and *A. actinomycetemcomitans* (Figure 4(a)), continuous biofilms with smooth surface were formed on both the blank and *β*-TCP groups. In the pure Mg group, bacteria were able to connect but did not form a continuous biofilm. Comparatively, the Mg-0.1Cu group showed few of the individual bacteria without formation of a biofilm. Similar results were observed in the multispecies biofilm model (Figure 4(b)). A large scale of smooth biofilm was only seen in the blank and *β*-TCP groups. The pure Mg group showed less biofilm formation, while the bacteria in the Mg-0.1Cu group lost connection with each other and the biofilm collapsed completely due to apoptosis of bacteria.

### 3.5. Transmission Electron Microscope Analysis

**Figure 5:**
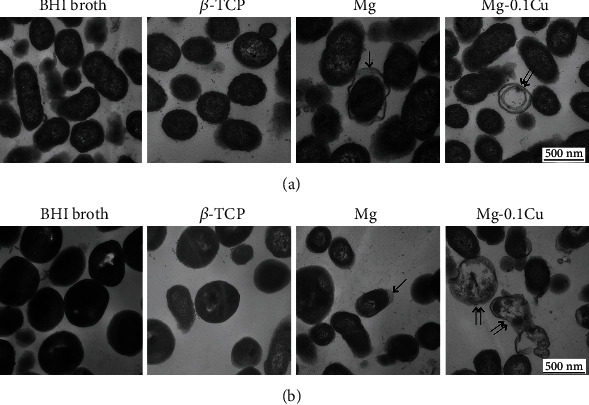
TEM images of the cell membrane and structure of Pg (a) and Aa (b) after culturing in extracts and BHI broth for 24 h, scale bar = 500 *μ*m. Single arrow shows the change of bacterial membrane in the Mg group. The cell membrane showed folds and stratification in the pure Mg group, where the membrane was separated from cytoplasm. Double arrows show the bacteria change in the Mg-Cu alloy group. The dead bacteria showed almost intact cell membranes with little cytoplasm remaining.

TEM observation showed the change of cell membranes and structures of *P. gingivalis* and *A. actinomycetemcomitans* after coculturing in different groups for 24 h (Figure 5). In blank and *β*-TCP groups, the integrity of the bacterial cells was unchanged, and bacterial cytoplasm was clear. Comparatively, the cell membrane showed folds and stratification in the pure Mg group, where the membrane was separated from the cytoplasm, while in the Mg-0.1Cu group, the dead bacteria showed almost intact cell membranes with little cytoplasm remaining.

## 4. Discussion

This study was designed to explore a potential treatment strategy for periodontal disease with novel Mg-Cu grafts which possessed the antibacterial ability against *P. gingivalis* and *A. actinomycetemcomitans*.

Periodontal bone grafting is a widely used surgery to promote periodontal regeneration, while inflammation control after surgery is an intricate problem in the clinic. Since bone substitutes have no antimicrobial properties, routine use of antibiotics was reported for guided bone regeneration by 97% of the survey respondents, with penicillin and doxycycline which are frequently used. The antibiotic uses before and after surgery with time length vary from 7 days to 10 days [35]. However, antibiotic abuse is currently a global challenge since the number of bacterial strains that are resistant to multiple types of antibiotics has dramatically increased each year worldwide [36]. In that case, it is urgent to find an alternate to reduce the use of antibiotics after periodontal surgery.

The Mg-Cu alloy which is considered to inhibit bacteria growth was proposed and investigated systematically in previous studies [22, 37]. It has been proven that Cu alloying in Mg exhibited long-lasting antibacterial effect against *Staphylococcus aureus* by the synergistic effects of Mg degradation and Cu ion release [21]. Furthermore, there was no cytotoxicity to both HUVECs and MC3T3-E1 cells during the degradation of the Mg-Cu alloy, and the proper release of Mg and Cu ions provided favorable promotion to osteogenesis and angiogenesis, indicating a great potential in bone grafting application. However, most PD-related bacteria are anaerobic, which have different growth characteristics from *Staphylococcus aureus*. In order to prove the efficacy of the Mg-Cu alloy on periodontal pathogens, anaerobic bacteria such as *P. gingivalis* and *A. actinomycetemcomitans* were selected in this study.

The pH value was positively correlated to the Cu content in the Mg-Cu alloy (Figure 1(a) due to severe galvanic corrosion. The pH value for the pure Mg extract was the lowest followed by Mg-0.1Cu, Mg-0.2Cu, and Mg-0.4Cu alloys in sequence. The survival of most PD-related bacteria depends on the pH homeostasis, since most proteins including enzymes only behave their functions in a neutral range of pH values. Exposure to a higher alkaline environment would pose stress on the bacterial cytoplasmic pH homeostasis [38]. Other studies also implied the antibacterial properties of Mg and its alloys [13, 14]. However, the present study raised two drawbacks of pH enhancement-induced antibacterial effect. First, when an Mg alloy device is implanted, change of the pH value will easily be balanced by body fluid. Therefore, the antibacterial ability of the Mg alloy *in vivo* is questionable. When the increase of pH is balanced, the antibacterial property would disappear as demonstrated in the previous study [18]. Second, high alkalinity circumstance may only switch the normal bacteria to a nonmetabolically active state. Many studies have proven that bacteria could survive in the extreme environment by switching to inactive status [39, 40]. In this study, the live/dead imaging (Figure 3) also indicated that high percentages of both *P. gingivalis* and *A. actinomycetemcomitans* were still alive in the pure Mg group.

Due to the drawbacks mentioned above, the introduction of antimicrobial metal elements such as Ag and Cu into the Mg alloy has gained more attention [37, 41]. Cu ion is known to cause multiple toxic effects such as generation of reactive oxygen species, lipid peroxidation, protein oxidation, and DNA degradation [42]. With higher content of Cu, more Cu ions are eluted from the implant material and bonded to the surface of bacteria, which eventually damage the cell membranes and kill the bacteria. Cu ions can hardly be balanced by human body fluid nor diluted by surrounding medium after releasing from the implant. Thus, the antibacterial activity will be maintained for the Mg-Cu alloy after implantation *in vivo*. In addition, the live/dead staining demonstrated that the release of Cu ions led to the necrosis of both *P. gingivalis* and *A. actinomycetemcomitans* (Figure 3) instead of switching them to the inactive state. The TEM observation showed that only the bacteria membrane changed in the pure Mg group, while the cytoplasm was lost completely in the Mg-Cu group with almost intact membranes. This indicates that the antibacterial mechanism of Cu should be different from that of the high pH value. It has previously been shown that Cu ions exert an antimicrobial activity, leading to increased influx of Cu^2+^ into bacteria and generation of reactive oxygen species, which results in inhibition of respiration and degradation of DNA and loss of cytoplasm [43].

The biofilm is critical for the microbial to attach to the material surface for survival [44, 45]. Both *P. gingivalis* and *A. actinomycetemcomitans* were not able to form a continuous biofilm as single species or multispecies (Figure 4) and were only observed as individual cells when incubated in the Mg-Cu alloy extracts. Without the protection from the biofilm, the cell membrane was destroyed directly and all the intracellular structures leaked outside (Figure 5), leading to bacterial apoptosis eventually. Another study also showed that on the surface of a Cu-implanted alloy, the shapes of *Escherichia coli* and *Staphylococcus aureus* changed obviously and their membranes were damaged [46]. Thus, Cu played a very important antibacterial role in the Mg-Cu extracts.

In the previous study [21], it has been demonstrated that the Mg-Cu alloy with the lowest Cu content could also enhance the cell viability, alkaline phosphatase activity, and osteogenesis-related gene and protein expressions. Thus, the biodegradable Mg-Cu alloy with osteogenesis, angiogenesis, and favorable antibacterial ability to PD-related bacteria is considered as a promising bone graft in periodontal regeneration. It is supposed that the Mg-Cu bone grafts in the form of particles can be filled into the alveolar bone pocket to treat the periodontal disease and enhance the possible regeneration of alveolar bone as well. The antibacterial and osteopromotive properties of Mg-Cu grafts will be further verified *in vivo* on a clinical relevant PD infection animal model.

## 5. Conclusions

The results in this study showed that with increase of the Cu content in the Mg-Cu alloy, the survival rates of both *P. gingivalis* and *A. actinomycetemcomitans* in the Mg-Cu alloy extracts were decreased significantly, and the bacteria cells and bacterial biofilms were destroyed obviously. The results are in agreement with other studies on Cu-bearing metals, indicating that the Mg-Cu alloy as potential bone grafts can effectively prevent infections in periodontal surgery.

## Data Availability

I have published all the data in the paper on Figshare (https://doi.org/10.6084/m9.figshare.12587297.v1).
